# 

**Published:** 1850-11

**Authors:** 


### CONTENTS

OF THE

### MEDICAL EXAMINER.

NEW SERIES—NO. LXXI.—NOVEMBER, 1850.

### ORIGINAL COMMUNICATIONS.

Observations on the Emmenagogue properties of Polygala Senega. By Caspar Morris, M. D., of Philadelphia, - - - - 627

Gastrotomy—(A case of Ovarian Dropsy, removal of sac and fatal termination.) By A. H. Grimshaw, M. D., of Wilmington, Del., Member of the American Medical Association. - - 630

Hospital Cases. Reported by J. M. Steiner, M. D,, Assistant Surgeon U. S. Army, Fort Graham, Texas. - - - - 634

Case of Osseous Deposit within the nervous pulp of a Molar Tooth. By S. S. Hornor, Dentist, - - - - - - 637

Case of Extra-Uterine Pregnancy, of over twenty years standing. By Wm. D. Christian, M. D., of Appomattox Co., Virginia, - 638

Case of Poisoning by the berries of the Swamp Dogwood. Reported by W. W. Johnson, N. C., Student of Medicine, - - 641

Wills' Hospital—Service of Dr. John Neill. Cases discharged from July 1st to Oct. 1st, 1850, - - - - - 642
Pennsylvania Hospital—Surgical Wards—Service of Dr. Norris. Cases discharged from Dec. 1st, 1849, to Jan. 1st, 1850, - 645

### BIBLIOGRAPHICAL NOTICES.

Observations on certain of the Diseases of Young Children. By Chas. D. Meigs, M. D., &c. &c., - - - - - 646
A Treatise on the Diseases and Physical Education of Children. By John Eberle, M. D., &c. &c. Fourth edition with cuts, and large additions, by Thomas D. Mitchell, M. D., &c. &c., - 646
A brief history of an existing controversy on the subject of Assimilated Rank in the Navy of the United States. By W. S. W. R., - 657

### EDITORIAL.

Southern Medical Students, - - - - - 662
Southern Medical and Surgical Journal, - - - - 664
Appointment of Dr. E. N. Horsford, - - - - 664
Appointment of Dr. Samuel Jackson, late of Northumberland, - 664
Deaths in Philadelphia from Sept. 21st to Oct. 19th, 1850. Reported by M. James Aitken Meigs, Student of Medicine, - - 665

### RECORD OF MEDICAL SCIENCE.

### ANATOMY AND PHYSIOLOGY.

A Retrospect of the Progress of Microscopic Investigation, and of the more important recent Contributions to Normal and Pathological Histology. By Robert D. Lyons, M. B., T. C. D., L. R. C. S. I., &c. &c., - - - - - 666
On the Structure of the Membrana Tympani in the Human Ear, - 673

### PATHOLOGY AND PRACTICE OF MEDICINE.

Peritonitis from Injury—Recovery, - - - - - - 674
Hay Fever—Its Cause, and its Cure by Nux Vomica, - - 677

Incontinence of Urine in Children. By J. Simon, Esq., F. R. S., Surgeon to St. Thomas's Hospital, - - - - - 678
Illustrations of the Difficulties which beset the Diagnosis of some Cases of Disease. By Thomas Poyser, Fellow of the Royal College of Surgeons of England, - - - - - 679

SURGERY.

Popliteal Aneurism; Tying of the Femoral Artery; Recovery. A New Aneurism-Needle, - - - - - - 682
Obliterated Brachial Artery, with Compound Fracture of the Humerus, - - - - - - - 684

